# Insights from the Genome Sequence of *Acidovorax citrulli* M6, a Group I Strain of the Causal Agent of Bacterial Fruit Blotch of Cucurbits

**DOI:** 10.3389/fmicb.2016.00430

**Published:** 2016-04-06

**Authors:** Noam Eckshtain-Levi, Dafna Shkedy, Michael Gershovits, Gustavo M. Da Silva, Dafna Tamir-Ariel, Ron Walcott, Tal Pupko, Saul Burdman

**Affiliations:** ^1^Department of Plant Pathology and Microbiology and the Otto Warburg Center for Agricultural Biotechnology, The Robert H. Smith Faculty of Agriculture, Food and Environment, The Hebrew University of JerusalemRehovot, Israel; ^2^Department of Cell Research and Immunology, George S. Wise Faculty of Life Sciences, Tel Aviv UniversityTel Aviv, Israel; ^3^Department of Plant Pathology, The University of Georgia, AthensGA, USA

**Keywords:** *Acidovorax citrulli*, bacterial fruit blotch, pathogenomics, horizontal gene transfer (HGT), pathogenicity, cluster of orthologs (COGs)

## Abstract

*Acidovorax citrulli* is a seedborne bacterium that causes bacterial fruit blotch of cucurbit plants including watermelon and melon. *A. citrulli* strains can be divided into two major groups based on DNA fingerprint analyses and biochemical properties. Group I strains have been generally isolated from non-watermelon cucurbits, while group II strains are closely associated with watermelon. In the present study, we report the genome sequence of M6, a group I model *A. citrulli* strain, isolated from melon. We used comparative genome analysis to investigate differences between the genome of strain M6 and the genome of the group II model strain AAC00-1. The draft genome sequence of *A. citrulli* M6 harbors 139 contigs, with an overall approximate size of 4.85 Mb. The genome of M6 is ∼500 Kb shorter than that of strain AAC00-1. Comparative analysis revealed that this size difference is mainly explained by eight fragments, ranging from ∼35–120 Kb and distributed throughout the AAC00-1 genome, which are absent in the M6 genome. In agreement with this finding, while AAC00-1 was found to possess 532 open reading frames (ORFs) that are absent in strain M6, only 123 ORFs in M6 were absent in AAC00-1. Most of these M6 ORFs are hypothetical proteins and most of them were also detected in two group I strains that were recently sequenced, tw6 and pslb65. Further analyses by PCR assays and coverage analyses with other *A. citrulli* strains support the notion that some of these fragments or significant portions of them are discriminative between groups I and II strains of *A. citrulli*. Moreover, GC content, effective number of codon values and cluster of orthologs’ analyses indicate that these fragments were introduced into group II strains by horizontal gene transfer events. Our study reports the genome sequence of a model group I strain of *A. citrulli*, one of the most important pathogens of cucurbits. It also provides the first comprehensive comparison at the genomic level between the two major groups of strains of this pathogen.

## Background

The *Acidovorax* genus belongs to the Betaproteobacteria class and comprises a variety of species with different lifestyles and inhabiting different environments. Some members of this genus are successful plant pathogens capable of infecting a wide range of agriculturally important crops (Rosenberg et al., 2015). Among these, *Acidovorax citrulli* (formerly *Acidovorax avenae* subsp. *citrulli*), has been the most investigated bacterium in recent years (Burdman and Walcott, 2012). This seedborne bacterium causes bacterial fruit blotch (BFB) of cucurbits. BFB gained importance after the occurrence of devastating outbreaks in watermelon fields in the Mariana Islands and the USA during the late 1980s and early 1990s (Latin and Rane, 1990; Somodi et al., 1991; Schaad et al., 2003). Since then, the pathogen has spread worldwide, mainly via contaminated seed, and was found to infect other cucurbit hosts, such as, melon, squash, pumpkin and cucumber (Bahar and Burdman, 2010; Burdman and Walcott, 2012). To date, there are no reliable sources of genetic disease resistance to BFB in the cucurbit germplasm, and chemical control has limited efficacy for disease management (Burdman and Walcott, 2012). Due to these reasons, and to the highly destructive potential of BFB, *A. citrulli* represents a serious threat to the cucurbit industry worldwide (Latin and Hopkins, 1995; Burdman and Walcott, 2012).

Most *A. citrulli* strains can be divided into two well-differentiated groups based on DNA fingerprinting, multilocus sequence analysis of housekeeping genes and fatty acid methyl ester profiles (Walcott et al., 2000, 2004; Burdman et al., 2005; Feng et al., 2009). Group I strains have been mainly isolated from melon and other non-watermelon cucurbits, while group II strains have been mainly isolated from watermelons. Recently, we showed that groups I and II strains of *A. citrulli* can be clearly distinguished based on differences in the arsenal and sequences of type III-secreted virulence effectors (Eckshtain-Levi et al., 2014).

In 2007, the Joint Genome Institute released the sequence of strain AAC00-1 (GenBank accession NC_008752), considered by the *A. citrulli* research community as the group II model strain of this bacterium. *A. citrulli* M6 was isolated in Israel in 2002 from a symptomatic melon fruit (Burdman et al., 2005) and in recent years has become the model group I strain for fundamental investigation of BFB. Using this strain we identified pathogenicity and virulence determinants of *A. citrulli*, including type III secretion (Bahar and Burdman, 2010), type IV pili (Bahar et al., 2009a, 2010) and polar flagella (Bahar et al., 2011). We also used M6 to characterize phenotypic variation in *A. citrulli* strains (Shrestha et al., 2013) and to develop PCR-based seed heath testing assays (Bahar et al., 2008), screen for BFB tolerance (Bahar et al., 2009b) and develop disease management strategies in cucurbit seedling production facilities (Chalupowicz et al., 2015).

Here we report the complete genomic sequence of *A. citrulli* strain M6. Comparative genome analyses reveal that the M6 genome is substantially shorter than that of AAC00-1. This mainly stems from the presence of eight fragments in AAC00-1 that are absent in M6. Importantly, we provide data supporting that most of these genomic differences are genetic markers that distinguish group I and II strains. GC content, effective number of codon (ENC) values and cluster of orthologs (COGs) analyses support the hypothesis that these fragments were introduced into *A. citrulli* group II strains by horizontal gene transfer (HGT) events.

## Results and Discussion

### Overview and Annotation of the *Acidovorax citrulli* M6 Genome

MiSeq sequencing of the *A. citrulli* M6 genome yielded 7.9 million high quality filtered reads of 150-bp average read length for paired-end and 4.4 million reads for mate-pair. Assembly was performed using the CLC Genomics Workbench, yielding 139 contigs with at least 70X coverage. The contigs had an average coverage of 270X, N50 was 170 kb and the average contig length was 34.6 kb. Assembly was facilitated by optical mapping (Schwartz et al., 1993) of the M6 draft genome, using the restriction enzyme KpnI and the genome of *A. citrulli* AAC00-1 (GenBank accession NC_008752) as a reference. Based on the OpGen MapSolver v.3.2.0 software estimation, the approximate size of the M6 genome is 4.85 Mb (**Table 1**). In agreement with this estimation, the sum of the 139 assembled contigs with 70X minimal coverage yielded 4,821,870 bp.

**Table 1:** General properties of the *Acidovorax citrulli* M6 genome.

Feature	M6	AAC00-1^a^
*A. cirulli* group	I	II
Size (Mb)	4.85	5.35
No. of contigs	139	1
Plasmids	0	0
Percent G + C content (%)	68.87	68.53
No. of ORFs	4,368	4,937
No. of shared ORFs	4,245	4,405
(by bi-directional hit/uni-directional hit)	(4,183/62)	(4,183/222)
No. of unique ORFs^b^	123	532
Average gene size (bp) {±standard error of the mean}	1,003 ± 10.3	1,014 ± 10.4
No. of RNA genes	51	62
No. of tRNA genes	48	53

*^a^Details of the *A. citrulli* AAC00-1 genome are provided for comparative purposes. The AAC00-1 genome was sequenced by the Joint Genome Institute and its sequence is available under GenBank accession NC_008752. For comparative purposes, ORFs of AAC00-1 were determined by RAST annotation (as similar as for M6).*

*^b^An ORF is unique if it is present in one strain and absent in the other.*

The 139 M6 contigs were annotated using RAST. The genome of strain M6 is comprised of a single chromosome without any detectable plasmids, following analysis with PlasmidFinder 1.3 (Carattoli et al., 2014). It has a relatively high G + C content of ∼68.9%. The high G + C content and the lack of plasmids is in agreement with the data from the AAC00-1 genome and genomes of other *Acidovorax* species in the public database (Byrne-Bailey et al., 2010; Xie et al., 2011; Ohtsubo et al., 2012). A total of 4,368 open reading frames (ORFs) were predicted with an average ORF length of 1,003 bp. Additional features of the M6 genome are summarized in **Table 1**. To verify the quality of the assembly and annotation, we performed tBlastN of 31 proteins universally distributed in bacteria (Wu and Eisen, 2008), and confirmed their presence in the assembled M6 genome (Supplementary Table S1). This whole genome shotgun project has been deposited at DDBJ/EMBL/GenBank under the accession LKUW00000000. The version described in this paper is version LKUW01000000.

### Comparative Analysis of the M6 and AAC00-1 Genomes

The M6 genome is substantially shorter than that of AAC00-1: 4.85 versus 5.35 Mb, respectively (**Table 1**). Different ORF prediction tools may yield different numbers of ORFs for the same assembly (Nielsen and Krogh, 2005; Ederveen et al., 2013). Therefore, although the annotation of the AAC00-1 genome is available at NCBI, for comparative purposes we annotated it using RAST, the same tool used for the M6 annotation. Indeed, the RAST annotation yielded a higher number of ORFs for AAC00-1 than predicted in NCBI. Notably, in the last version of the AAC00-1 genome available at NCBI (dating from July 30, 2015) some genes that were previously annotated based on their homology to known genes in other bacteria are now absent. For example, genes encoding the type III-secreted effector genes *Aave_2708* and *Aave_2938* (gene names according to older annotations of AAC00-1; Eckshtain-Levi et al., 2014) are missing in the new annotation.

**FIGURE 1:**
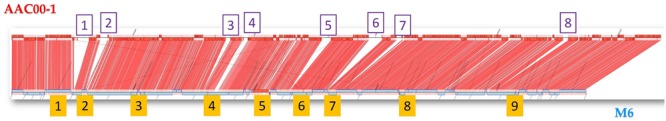
**Synteny between the genomes of *Acidovorax citrulli* strains M6 **(bottom)** and AAC00-1 **(top)** using CONTIGuator (Galardini et al., 2011).** The eight boxes at the **(top)** represent the 8 AAC00-1 DNA fragments that are absent in the M6 genome (FA fragments). The nine boxes at the **(bottom)** represent the reference fragments (RFAs) that were used as controls for coverage analyses of various *A. citrulli* strains. Length and nucleotide positions of FA and RFA fragments in the AAC00-1 genome are detailed in Supplementary Table S2.

Unidirectional BLAST analysis revealed that 97% of the M6 genes were present in the AAC00-1 genome, while 89% of the AAC00-1 genes were identified in the M6 genome (**Table 1**). If we consider only bidirectional hits, the percentages of shared ORFs drop to 95 and 85%, respectively. These differences are in agreement with the estimated genome sizes of the two strains. Compared to M6, the AAC00-1 genome has over 500 unique genes, most of which are located in eight fragments (hereafter FA1–FA8; for fragments of AAC00-1) ranging in size from ∼34.9 to ∼119.5 kb, and scattered throughout the genome (**Figure 1**; Supplementary Table S2). The presence and absence of these fragments in AAC00-1 and M6 genomes, respectively, explain the ∼ 500 kb difference in genome size between the two genomes. In order to verify this result we designed primer sets from randomly selected regions of the AAC00-1 FAs. Primer sequences and their targets in the AAC00-1 genome are shown in Supplementary Table S3. These primers were designed to produce PCR amplicons ranging from ∼900 to ∼1,500 bp in size (Supplementary Table S4), based on the AAC00-1 annotation. PCR assays were conducted using genomic DNA of strains AAC00-1 and M6 as well as an additional group II strain, 7a1 (Eckshtain-Levi et al., 2014). As expected, all primer sets yielded PCR products of expected sizes when DNA of strain AAC00-1 was used as template (Supplementary Figure S1A). In contrast, none of the primer sets for the eight AAC00-1 FAs produced amplicons from M6 genomic DNA (Supplementary Figure S1B). As a positive control, primers that target the housekeeping gene *gltA* (Eckshtain-Levi et al., 2014) were used, and a PCR amplicon of the expected size was produced with genomic DNA from strain M6 (Supplementary Figure S1B). These results are in line with the bioinformatics analysis that indicated that the eight FAs from the AAC00-1 genome are absent in the M6 genome. Similar to AAC00-1, PCR with genomic DNA from an additional group II strain, 7a1, yielded PCR products for all FA primer sets (Supplementary Figure S1B).

The M6 genome possesses 123 ORFs that are absent in AAC00-1 (**Table 1**). Based on the automated Blast results from the RAST server, the majority of these ORFs (96/123; 78.0%) fall into the category of hypothetical proteins. To search for possible functions of these ORFs, they were subjected to manual Blast Pin the NCBI server. For 24 of these ORFs we detected homologies to known proteins, reducing the percentage of hypothetical proteins to 58.5% (72/123; Supplementary Table S5). Interestingly, among the M6 genes that are absent in AAC00-1, at least seven were found to encode type IV secretion (T4S) system and conjugative transfer proteins (*APS58_01125, APS58_04005, APS58_05855, APS58_05885, APS58_07480, APS58_07485*, and *APS58_13215*; gene names according to the GenBank annotation). Based on the annotation of AAC00-1, the genome of this strain does not possess T4S genes. Other interesting genes present in M6 and absent in AAC00-1 included few encoding transport proteins such as *APS58_11385* and *APS58_11405*, both encoding Resistance-Nodulation-Division (RND) transporters, *APS58_00180*, encoding a putative cobalt ATP-binding cassette (ABC) transporter permease, and *APS58_10910*, encoding a lead/cadmium/zinc/mercury transporting ATPase (Supplementary Table S5).

The M6 genome also contains a gene (*APS58_00685*) encoding calpastatin, which is not annotated in AAC00-1. Calpastatin is an inhibitor of proteases that belong to the calpain family. In contrast to mammals where the calpain family is expanded, only single calpain genes are present in plants (Croall and Ersfeld, 2007). Studies on maize, tobacco and *Arabidopsis* support that phytocalpain plays a critical role in growth regulation and development in plants (Lid et al., 2002, 2005; Ahn et al., 2004; Johnson et al., 2008). While calpastatin genes are present in several plant-associated bacteria such as rhizobia and *Pseudomonas syringae*, to the best of our knowledge, their role in plant-microbe interactions have not been investigated. It will be interesting to assess whether the calpastatin gene has a role in virulence of *A. citrulli*.

The draft genome sequences of two *A. citrulli* strains isolated in China, tw6 and pslb65, were recently deposited in the GenBank database under accession numbers JXDJ00000000 and JYHM00000000, respectively. The approximated genome sizes of these strains are ∼4.9 Mb and ∼5.1 Mb for pslb65 and tw6, respectively. In the corresponding genome report manuscripts, strain pslb65 was reported as a group I strain (Wang et al., 2015a), while the group belonging of strain tw6 remained undetermined (Wang et al., 2015b). We generated a phylogenetic tree with sequence data from seven housekeeping genes (Yan et al., 2013; Eckshtain-Levi et al., 2014), which clearly clustered pslb65 and tw6 into group I (Supplementary Figure S2). We used BlastN to assess the presence and coverage of the 123 M6 ORFs that are absent in AAC00-1, in the genomes of these strains (Supplementary Table S5). A high coverage of these genes was found in the genome of strain pslb65: in fact, only 2 of the 123 ORFs (1.6%), corresponding to a hypothetical protein (APS59_00190) and a putative phage protein (APS58_10370), were not detected in the pslb65 genome. The coverage of these ORFs was lower in the genome of strain tw6; yet, the majority of these M6 genes (72/123; 60.2%) were present in this strain. Among the aforementioned T4S/conjugative transfer and transport ORFs present in the M6 genome and absent in AAC00-1, all were detected in the genome of pslb65 but only two of them, *APS58_13215* and *APS58_10910*, were present in the tw6 genome (Supplementary Table S5). The *APS58_00685* gene encoding calpastatin was detected in both pslb65 and tw6.

### The Eight AAC00-1 FAs Differentiate Group II from Group I Strains

**FIGURE 2:**
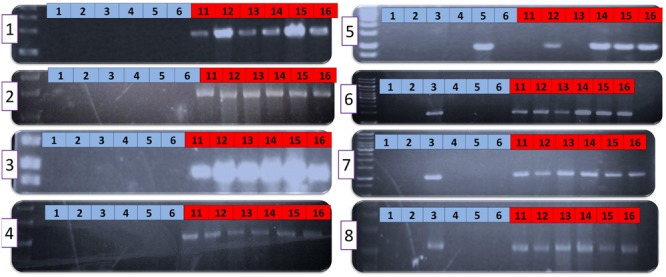
**PCR with primers targeting the eight FA fragments of selected groups I and II *Acidovorax citrulli* strains.** Numbers (1–8) inside purple-outlined boxes at the left of each gel represent the FA number. Blue and red boxes at the top of each gel correspond to DNA from groups I and II strains, respectively. Group I strains: 1, AAC98-17; 2, AAC200-23; 3, AACAU-9; 4, AAC200-30; 5, AAC92-305; 6, M6. Group II strains: 11, AAC94-48; 12, AAC201-20; 13, AAC94-55; 14, AAC202-69; 15, AAC94-87; 16, AAC201-19. Strain details are provided in **Table 2**.

To further explore whether the AAC00-1 FAs (FA1 – FA8) reflect universal differences between groups I and II strains of *A. citrulli*, we used the primer sets described above (Supplementary Table S3) to screen other representative groups I and II *A. citrulli* strains. The tested strains were isolated from different geographic locations and represent various *A. citrulli* haplotypes based on pulse field gel electrophoresis (PFGE) profiling (**Table 2**). DNA from most group II strains yielded PCR amplicons of expected sizes. The only exceptions were strains AAC94-48, AAC-94-55, W4 and W6 that did not yield a PCR product corresponding to FA5. In contrast, PCR reactions with DNA from representative group I strains did not yield PCR products for fragments FA1–FA4. The picture was more complex for fragments FA5–FA8. While most group I strains did not yield PCR products for these fragments, there were few exceptions: strain AAC92-305 yielded an amplicon for FA5, strain AAC200-23 yielded amplicons for FA6–FA8, and the sequenced strain pslb65 yielded amplicons for FA7 and FA8 (**Table 2**). Results of PCR assays conducted with genomic DNA from some of the strains are shown in **Figure 2**. All tested strains yielded PCR products for the control gene, *gltA* (not shown).

**Table 2:** *Acidovorax citrulli* strains tested by PCR analysis with sets of primers targeting regions from the AAC00-1 FA fragments^a^.

G	Strain	PFGE haplotype^b^	Country	FA1	FA2	FA3	FA4	FA5	FA6	FA7	FA8
**I**	AAC92-305^c^	B2 (I)	Unknown	-	-	-	-	+	-	-	-
	AACAU-9^d^	B5 (M)	Australia	-	-	-	-	-	-	-	-
	AAC98-17^c^	B6 (N)	USA	-	-	-	-	-	-	-	-
	AAC200-23^d^	B8 (P)	USA	-	-	-	-	-	+	+	+
	AAC200-30^d^	B10 (S)	USA	-	-	-	-	-	-	-	-
	M1^e^	B21	Israel	-	-	-	-	-	-	-	-
	M4^e^	B21	Israel	-	-	-	-	-	-	-	-
	M6^e^	B21	Israel	-	-	-	-	-	-	-	-
	5^f^	B21	Israel	-	-	-	-	-	-	-	-
	tw6^g^	n.d.	China	-	-	-	-	-	-	-	-
	pslb65^h^	n.d.	China	-	-	-	-	-	-	+	+

**II**	W1^e^	A1 (A)	Israel	+	+	+	+	+	+	+	+
	AAC201-19^d^	A2 (B)	Australia	+	+	+	+	+	+	+	+
	AAC201-20^d^	A3 (C)	Australia	+	+	+	+	+	+	+	+
	AAC00-1^c^	A1 (A)	USA	+	+	+	+	+	+	+	+
	AAC94-55^d^	A5 (E)	USA	+	+	+	+	-	+	+	+
	AAC94-87^d^	A6 (G)	USA	+	+	+	+	+	+	+	+
	AAC94-48^d^	A9 (U)	USA	+	+	+	+	-	+	+	+
	AAC202-69^d^	A11 (W)	Thailand	+	+	+	+	+	+	+	+
	W4^e^	A13	Israel	+	+	+	+	-	+	+	+
	W6^e^	A20	Israel	+	+	+	+	-	+	+	+
	7a1^f^	A23	Israel	+	+	+	+	+	+	+	+

*^a^FA numbers are according to their location in the sequenced strain AAC00-1 (**Figure 1**). For exact coordinates see Supplementary Table S2. G, *A. citrulli* group; +, positive PCR; -, negative PCR.*

*^b^Haplotypes based on PFGE analyses following digestion with *SpeI*. Where relevant, letters between brackets indicate the old haplotype designation as described in previous publications (Walcott et al., 2000, 2004). Due to the identification of new haplotypes in recent years, the haplotype classification was changed from single letters to combinations of a letter and a number, where A and B indicate groups II and I, respectively. n.d., not determined.*

*^c-h^Sources of bacterial strains: Walcott et al. (2000, 2004), Burdman et al. (2005), Eckshtain-Levi et al. (2014), Wang et al. (2015a,b), respectively.*

The PCR assays targeting the FAs strengthened the notion that, overall, the differences between AAC00-1 and M6 regarding the eight AAC00-1 FAs, apply globally to groups I and II *A. citrulli* strains. With regards to these findings, it is worth mentioning that gene *Aave_2708* (name according to the annotation of AAC00-1), encoding a type III-secreted effector belonging to the C55-family of cysteine proteases or serine/threonine acetyltransferases, which was present in all tested group II *A. citrulli* strains but absent in group I strains (Eckshtain-Levi et al., 2014), is located in FA6 of AAC00-1. Similarly, we are currently characterizing a *vapBC*-like toxin-antitoxin locus located in AAC00-1 FA1 that was detected in 12 of 12 group II strains but 0 of 15 group I strains. All strains tested represented different PFGE haplotypes (Shavit et al., 2016).

**FIGURE 3:**
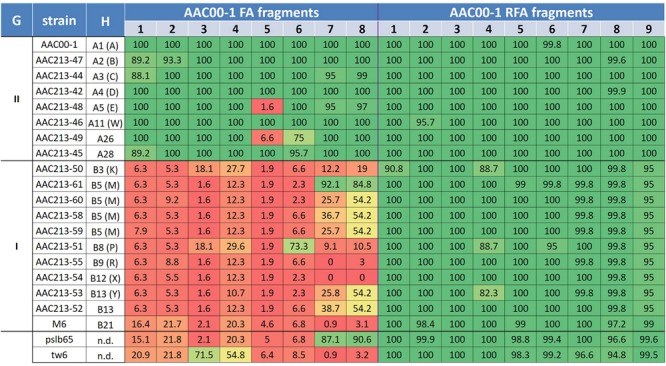
**Coverage percentage of *Acidovorax citrulli* strain AAC00-1 FA fragments 1–8 and RFA fragments 1–9 in the draft genomes of several group I and II *A. citrulli* strains.** The fragments were used as queries in MegaBlast analyses (Geneious 8.1.7) against the strain contigs and the total coverage was calculated. The percentage of coverage is indicated for each fragment/strain. To emphasize the picture, a color scale was used that correlates with the level of coverage. From low to high coverage: dark red, light red, orange, yellow, light green, dark green. G, group; H, Haplotype designation based on PFGE analysis following digestion with *SpeI*. Where relevant, letters between brackets indicate the old haplotype designation as described in previous publications (Walcott et al., 2000, 2004); n.d., not determined.

The aforementioned data indicate some level of specificity of the FA1–FA8 fragments to group II *A. citrulli* strains. However, we should be careful with this notion, since the primer sets tested in this study represent only a small portion of these fragments. We recently got access to the proprietary draft sequences of seventeen genomes of *A. citrulli* strains from several haplotypes (10 from group I; 7 from group II), sequenced by a private company (anonymity requested). We used MegaBlast to determine the percent coverage of the AAC00-1 FA1-FA8 fragments in the sequences of the aforementioned genomes, as well as of strains tw6 and pslb65. As controls, we arbitrarily selected nine reference fragments from the AAC00-1 genome (hereafter RFA1 to RFA9), ranging in size from 30.4 to 121.2 kb, and interspersed between fragments FA1–FA8 (**Figure 1**; Supplementary Table S2). The RFAs were highly conserved in all *A. citrulli* strains (**Figure 3**). In contrast to the picture observed for the RFAs, and in agreement with the PCR results reported above, this analysis supports a clear distinction between groups I and II strains in terms of coverage of the eight FAs (**Figure 3**). Also, in line with the phylogenetic analysis of housekeeping genes (Supplementary Figure S2), the sequenced strains from China, tw6 and pslb65, clustered with group I (**Figure 3**).

A clear distinction between group I and II strains was observed for fragments FA1 to FA4, which showed an overall high level of coverage in group II strains, and relatively low level of coverage in group I strains (**Figure 3**). One exception was strain tw6 that showed intermediate levels of coverage for fragments FA3 and FA4 (71.5 and 54.8%, respectively). A similar pattern was observed for most of the tested strains for fragments FA5–FA8. However, there were a few exceptions. For instance, in agreement with the PCR results for the group I strains AAC200-23 and pslb65 (**Table 2**), some group I strains showed intermediate to high levels of coverage (ranging from 54.2 to 92.1%) for fragments FA6–FA8 (**Figure 3**). As mentioned above, four group II strains did not yield a PCR product for fragment FA5 (**Table 2**). Coverage analysis revealed low coverage levels for this fragment for two group II strains, AAC213-48 and AAC213-49 (1.6 and 6.6%, respectively). In this regard, it is notable that AAC213-48 and AAC94-55 did not yield a PCR fragment for FA5, and both belong to the same PFGE haplotype (A5). In contrast, little discrepancies were observed between PCR and coverage analyses. For instance, based on PCR analysis, strain AAC200-23 (haplotype B8) possesses at least part of each of the FA6–FA8 fragments. However, while a relatively high coverage was observed for fragment FA6 for the haplotype B8 strain AAC213-51 (73.3%), relatively low coverage was found in this strain for fragments FA7 and FA8 (9.1 and 10.5%, respectively). Similarly, while the group I (haplotype B5) strain AACAU-9 did not yield PCR products for any FA fragment (**Table 2**), other group I strains belonging to the same haplotype showed intermediate to high levels of coverage for fragments FA7 and FA8 (**Figure 3**). Possible reasons for the above inconsistencies might be: (i) limited level of representation of the PCR tests (e.g., targeting a small portion of the FAs); and (ii) genetic variability between strains, even those that belong to the same haplotype. Nevertheless, despite these inconsistencies, there was a high level of agreement between the two approaches.

### Sequence Analyses of Fragments FA1 to FA8

**FIGURE 4:**
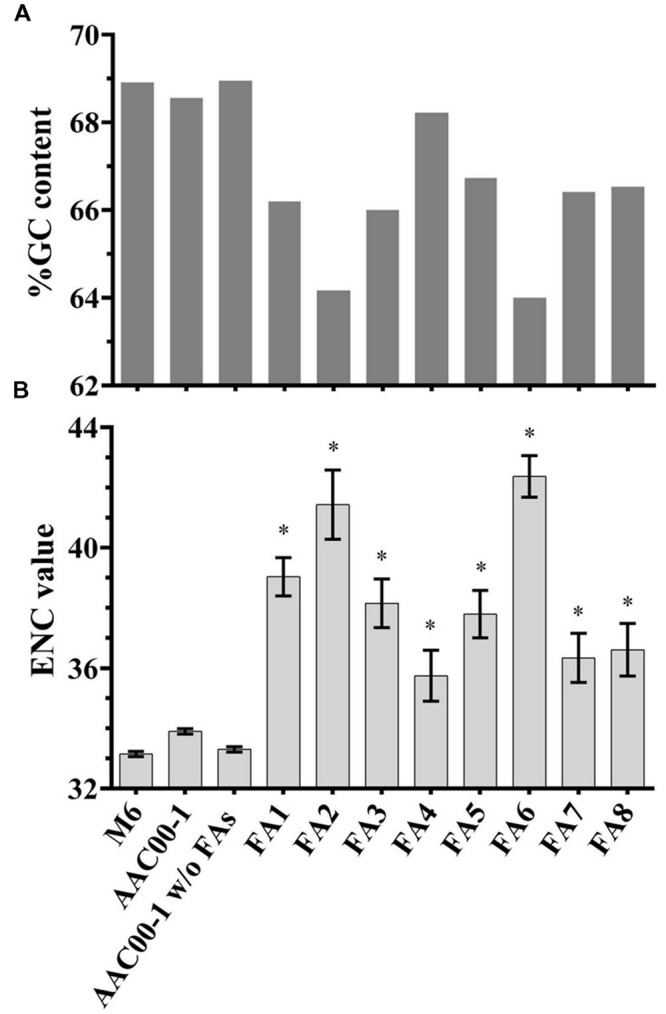
**G + C content and ENC value analyses for *Acidovorax citrulli* M6, AAC00-1, AAC00-1 without the FAs and each of the eight FA fragments. (A)** G + C content was calculated using GC-Profile web server; **(B)** ENC values of predicted ORFs were determined using BioPython package for Python. Data represent average ENC values and standard error. Asterisks above the FAs indicate that the ENC value significantly (*p* < 0.05) differs from the ENC value of AAC00-1 without the FAs according to student’s *t*-test with Bonferroni adjustment.

To gain insight into fragments FA1–FA8, we analyzed their G + C content, ENCs and COGs, as these features may indicate recent HGT events (Ochman et al., 2000; Philippe and Douady, 2003). As shown in **Table 1**, the G + C content of M6 is slightly higher than that of AAC00-1 (∼68.9% versus ∼68.5%, respectively). Interestingly, the G + C content of the AAC00-1 FA fragments was lower than that of the whole AAC00-1 genome (**Figure 4A**). Six fragments, FA1–FA3 and FA6–FA8, have G + C contents that are lower than 67%. Among them, FA2 and FA6 have G + C contents ranging from 63 to 64%. The other two fragments, FA4 and FA5, have higher G + C contents than the other FA fragments, but still lower than 67.5% (namely, more than 1% lower than the whole AAC00-1 genome; **Figure 4A**). Moreover, G + C content analysis of the AAC00-1 genome after exclusion of the FAs showed that it is almost identical to that of strain M6 (lower by less than 0.05%). This result implies that the FA fragments, that represent approximately 10% of the AAC00-1 genome, explain the slightly lower G + C content of the AAC00-1 genome relative to that of M6.

In order to assess whether differences in G + C content are statistically significant, we calculated the G + C content of each ORF in each FA. For each FA, we next compared the average G + C content of its ORFs with the average over all ORFs in the AAC00-1 genome, excluding the FAs. All but one fragment, FA4, had an average G + C content significantly (*t*-test; *p* < 0.05) lower than the average G + C content of the AAC00-1 genome excluding the FAs (Supplementary Figure S3).

ENC analysis provides an estimate of the codon usage biases in genes or genomes. ENC values may range from 20, where one codon is used exclusively for each amino acid, to 61, representing no codon bias (Wright, 1990; Behura and Severson, 2013). Genes or genome regions having ENC values higher than the whole genome may indicate recent acquisition by HGT (Guerdoux-Jamet et al., 1997; Ochman et al., 2000). While the average ENC value of all AAC00-1 ORFs was 33.9, that of M6 was 33.1. Analysis of the FAs revealed that the present ORFs have an average ENC of 38.8, which is substantially greater than the whole AAC00-1 genome. ENC values of all FAs were significantly (*t*-test; *p* < 0.05) higher than that of the AAC00-1 genome excluding these fragments (**Figure 4B**). This was also the case for FA4, which had the lowest average ENC value (35.7). The other seven FAs have average ENC values greater than 36, with fragments FA2 and FA6, having ENC values of 41.4 and 42.3, respectively (**Figure 4B**). As mentioned above, FA2 and FA6 also showed the lowest G + C contents (**Figure 4A**; Supplementary Table S2; Supplementary Figure S3). It is notable that for both G + C content and ENC analyses, the values obtained for AAC00-1 without the FAs are substantially closer to those of the M6 genome (**Figure 4**).

**FIGURE 5:**
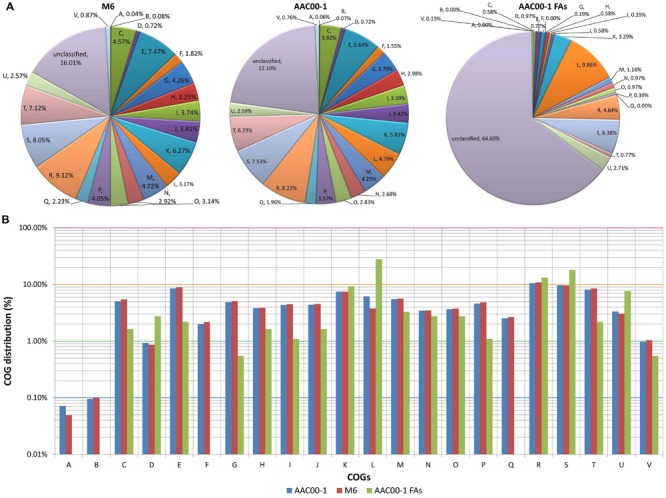
**COG distribution in *Acidovorax citrulli* M6, AAC00-1 and AAC00-1 FA fragments. (A)** Pie plot illustrating COG distribution of predicted ORFs from M6 and AAC00-1 genomes as well as from the eight AAC00-1 FA fragments. ORFs that could not be classified into any category are grouped as “unclassified.” **(B)** Column plot representation of COG distribution. The unclassified ORFs were excluded from this analysis. COG functional categories: (A) RNA processing and modification; (B) chromatin structure and dynamics; (C) energy production and conversion; (D) cell cycle control, cell division and chromosome partitioning; (E) amino acid transport and metabolism; (F) nucleotide transport and metabolism; (G) carbohydrate transport and metabolism; (H) coenzyme transport and metabolism; (I) lipid transport and metabolism; (J) translation, ribosomal structure and biogenesis; (K) transcription; (L) replication, recombination and repair; (M) cell wall/membrane/envelope biogenesis; (N) cell motility; (O) post-translational modification, protein turnover, chaperones; (P) inorganic ion transport and metabolism; (Q) secondary metabolites biosynthesis, transport and catabolism; (R) general functional prediction only; (S) function unknown; (T) signal transduction mechanisms; (U) intracellular trafficking, secretion, and vesicular transport; and (V) defense mechanisms.

We next analyzed differences in functional categories between the M6 and AAC00-1 genomes, focusing on the functions of genes encoded in the FA1-FA8 fragments. Overall, few differences were observed between AAC00-1 and M6 in COG distribution of predicted ORFs. Nevertheless, the AAC00-1 genome has a higher percentage of unclassified proteins (i.e., could not be classified to any COG category) than the M6 genome: 22.1% versus 16.0%. This is partially explained by the relatively high percentage (64.6%) of unclassified ORFs in the AAC00-1 FA fragments, which account for 502 predicted ORFs (**Figure 5A**).

In order to get a clearer picture, without the “disturbance” of unclassified ORFs, we plotted the classified ORFs alone in a semi-logarithmic graph (**Figure 5B**). This analysis showed that three COG categories are over-represented in the FA fragments, at over twofold relative to whole AAC00-1 and M6 genomes. These three categories are comprised of genes that are associated with HGT (Nakamura et al., 2004; Qiu et al., 2006; Juhas et al., 2008; Zhu et al., 2015) such as ATPases involved in chromosome partitioning in COG category D (cell cycle control, cell division, chromosome partitioning; 3.1-fold more in the FAs); transposases and integrases for COG L (replication, recombination and repair; 5.6-fold more in the FAs); and components from the type IV secretory pathway in COG category U (intracellular trafficking, secretion, and vesicular transport; 2.4- fold more in the FAs). Moreover, 43% of the genes found in the FAs and classified into COG L encode transposases and integrases, while most COG D members encode ATPases associated with chromosome segregation and have homology with the ParA family of proteins, which were shown to be required for maintenance of pathogenicity islands (Qiu et al., 2006).

In contrast to the above categories that are associated with HGT, COGs corresponding to general metabolism and other housekeeping mechanisms (Jain et al., 1999; Sorek et al., 2007; Xu et al., 2007) are under-represented in the FAs. Among those, the most under-represented was COG G (carbohydrate transport and metabolism; ninefold less than in complete AAC00-1 and M6 genomes). Other highly under-represented categories were COGs C (energy production and conversion), T (signal transduction), E (amino acid transport and metabolism), I (lipid transport and metabolism) and P (inorganic ion transport and metabolism) (3.2, 3.8, 4, 4, and 4.3-fold less than in overall genomes, respectively). Interestingly, genes from four COG categories were not detected in the eight FAs: A (RNA processing and modification), B (chromatin structure and dynamics), F (nucleotide transport and metabolism) and Q (secondary metabolites biosynthesis, transport and catabolism).

The combined data from comparative sequence analyses as well as G + C content, ENC and COG analyses strongly indicate a scenario by which the eight AAC00-1 FAs are typical of *A. citrulli* group II genomes, but rare in genomes of group I strains. Additionally, these fragments or a large portion of them have been recently introduced into group II strains via HGT events. We hypothesize that these fragments were gradually acquired by several HGT events by ancestral group I strains, leading to a separation of the two groups and their subsequent adaptation to different hosts in the *Cucurbitaceae* family. In support of this hypothesis, the first report of *A. citrulli* in the USA (Webb and Goth, 1965) involved group I strains (Burdman and Walcott, 2012). Webb and Goth (1965) described a disease in seedlings of two watermelon plant introductions originated in Turkey, in a Regional Plant Introduction Station (RPIS) at Georgia, USA. At that time, the pathogen was considered to only affect seedlings and have a low potential for damage on watermelon fruits in the field (Sowell and Schaad, 1979). The type strain of *A. citrulli* (ATCC 29625/C-42), isolated from the aforementioned occurrences at the Georgia RPIS (Schaad et al., 1978), was later determined to be PFGE haplotype B3 (K according to the old designation), which is in group I (Walcott et al., 2000, 2004). The destructive potential of *A. citrulli* was only recognized in 1987, when the first BFB outbreaks occurred in the Mariana Islands, leading to fruit infection/rot that translated into significant yield losses (Wall and Santos, 1988). In the following years, severe BFB outbreaks occurred in watermelon fields in the USA (Latin and Rane, 1990; Somodi et al., 1991). These outbreaks in watermelon fields in the USA, and later in other parts of the world, were shown to be caused by emerging group II strains (Walcott et al., 2000, 2004; Burdman and Walcott, 2012). Despite this, we cannot exclude the possibility that some of the FA fragments are unstable in group II genomes, and some may have been lost, partially or entirely, by some group II strains. The relative coverage of some FA fragments (e.g., FA6–FA8) in group I genomes and the low coverage of FA5 in a few group II haplotypes, suggest that both acquisition and loss events may have occurred. In this regard, both PCR and coverage analyses indicate that fragments FA1–FA4 are more discriminative between groups I and II strains than fragments FA5–FA8.

Overall, the above hypotheses as well as hypotheses regarding the general evolution of *A. citrulli* groups are very difficult to verify. We speculated that *A. citrulli*, and particularly group I strains, originated in Asia. In support of this notion, Yan et al. (2013) recently reported high frequency and genetic diversity of group I strains isolated in China. Furthermore, Feng et al. (2009) reported that the majority of *A. citrulli* strains assessed from China were members of MLST clonal complex 1 (that corresponds to PFGE group I). Despite this, there is a large lack of knowledge regarding the history of BFB and tracking of *A. citrulli* isolates from this part of the world. Moreover, since the early 1990s’ BFB has spread rapidly, both by groups I and II strains, to different cucurbits and to many parts of the world, mainly by contaminated seeds (Burdman and Walcott, 2012). Due to the globalization of seed trade, it is virtually impossible to determine the true origin of a given strain. Nevertheless, this study contributes important insights toward the understanding of the genomic differences between the two main groups of this threatening pathogen. It also provides new leads to investigate the genetic determinants of host preferential association of the two groups.

## Conclusion

Here we reported the genome sequence of strain M6, the group I model strain of *A. citrulli.* We also performed the first comprehensive genomic comparison between a group I strain and the group II model strain, AAC00-1. The M6 genome is shorter than the AAC00-1 genome, and this is mainly explained by the absence in M6 of eight DNA fragments that are present in AAC00-1. Further analyses of other groups I and II strains indicate that these fragments likely contain the genetic determinants that distinguish the two major groups of *A. citrulli*. We also provide evidence supporting the hypothesis that these fragments or a significant portion of them have been introduced into group II strains by HGT events. Further investigation is needed to elucidate the genetic determinants that distinguish groups I and II strains. Differences in host preference between strains of the same species, makes *A. citrulli* a unique model for the investigation of fundamental phytopathogenic bacteria–plant interactions.

## Material and Methods

### Bacterial Strains

*Acidovorax citrulli* strains used in this work are listed in **Table 2** and **Figure 3**. Bacteria were grown in nutrient broth (NB, Difco Laboratories, Detroit, MI, USA) or NA (NB containing 15 g/l agar) at 28°C.

### Sequencing of the M6 Genome

Bacterial DNA was isolated with the GenElute^TM^ Bacterial Genomic DNA Kit (Sigma-Aldrich, St. Louis, MO, USA) according to the manufacturer’s instructions. Genomic DNA was prepared for sequencing using the Illumina Nextera XT kit and the Nextera Mate Pair Sample Preparation Kit (Illumina, Inc., San Diego, CA, USA) according to the manufacturer’s instructions. The gel-free protocol was employed for the mate pair library. After purification, the library was pooled in an approximately equimolar ratio, and quantified using the KAPA Library Quantification Kit-Illumina (KAPA Biosystems, Woburn, MA, USA). Library preparation was performed at the DNA Services Facility at the University of Illinois (Chicago, IL, USA) and sequencing was performed by an Illumina MiSeq instrument, employing paired-end 150-base reads at the W.M. Keck Center for Comparative and Functional Genomics (University of Illinois, Urbana, IL, USA). Standard paired-end library generated approximately 7.9 M reads per sample (paired), and approximately 4.4 M reads per sample were generated for mate-pair library.

### Assembly and Annotation of the M6 Genome

After quality trimming and PhiX removal (Quail et al., 2008), trimmed reads were assembled by the *de novo* assembler within the software package CLC Genomics Workbench v 7.0^1^ (CLCbio, Cambridge, MA, USA). Optical mapping of the M6 genome was performed by OpGen (OpGen, Inc., Gaithersburg, MD, USA), using Whole Genome Mapping (WGM) technology, as described (Miller, 2013) using the restriction enzyme KpnI. The whole genome map was compared to the *de novo* assembly using the software package MapSolver v.3.2.0^2^. One hundred thirty nine contigs, with 70X coverage and above, were uploaded and annotated using RAST web server^3^ (Aziz et al., 2008; Overbeek et al., 2014; Brettin et al., 2015). In order to compare ORF prediction between genomes with the same parameters, the AAC00-1 genome sequence (GenBank NC_008752) was also annotated using RAST. The RAST annotations were validated with Prodigal (Hyatt et al., 2010), which yielded similar results. As an additional quality control we tested the M6 sequence for the presence of 31 housekeeping genes that are universally conserved in bacteria. The sequences of the corresponding proteins were collected from *Escherichia coli* K12. The amino acid sequences of the ortholog genes were retrieved from AAC00-1 using tBlastN and used to screen the contigs of the M6 genome by tBlastN. All BLAST analyses were done using the BioCyc website^4^ (Caspi et al., 2014).

### Sequence Analysis Tools

The M6 genome was aligned to AAC00-1 using the CONTIGuator web server (Galardini et al., 2011). Screening for plasmids in M6 sequence was done using PlasmidFinder 1.3 (Carattoli et al., 2014). tRNA genes were detected using the tRNAScan-SE program (Pavesi et al., 1994). G + C content was calculated using the GC-Profile web server (Gao and Zhang, 2006). ENC values and G + C content of ORFs in M6 and AAC00-1 genomes as well as in the AAC00-1 FAs were calculated using the BioPython package (Cock et al., 2009) for Python^5^ (Oliphant, 2007). Clusters of orthologs groups (COGs) were assigned to each predicted protein using the WebMGA web server (Altschul et al., 1990; Wu et al., 2011). Coverage analyses of the AAC00-1 FAs and control fragments (RFAs) in the draft genome sequences of *A. citrulli* strains were performed with the MegaBlast program implemented on Geneious version 8.1.7 (Biomatters Ltd., Auckland, New Zealand^6^), using the following parameters: maximum e-value, 0.0001; gap cost, linear; match-mismatch scoring, 1-2; maximum hits, 100. To verify the group of the recently sequenced *A. citrulli* strains from China, tw6 and pslb65 (Wang et al., 2015a,b), a phylogenetic tree based on partial sequences of seven housekeeping genes (*gltA, trpB, lepA, ugpB, gmc, phaC*, and *pilT*) was generated using the sequences of these strains and of other *A. citrulli* strains for which these sequences are available in the NCBI database (Feng et al., 2009; Eckshtain-Levi et al., 2014). The tree was generated as previously described (Eckshtain-Levi et al., 2014). Briefly, the sequences were aligned using the MAFFT software (Katoh and Standley, 2013) and a maximum likelihood tree was generated using MEGA6 software (Tamura et al., 2013). Bootstrap values were derived from 1,000 replicates in each case to validate tree topology. The outgroup consisted of ortholog sequences from the closely related *Acidovorax avenae* ICPB 30003.

### Polymerase Chain Reaction (PCR)

To assess if the AAC00-1 FA fragments were present in the genomes of several, non-sequenced *A. citrulli* strains, we designed sets of primers corresponding to each fragment using Primer3 v.0.4.0 (Koressaar and Remm, 2007; Untergasser et al., 2012), based on the AAC00-1 sequence. PCR primers, listed in Supplementary Table S3, were purchased from Hy Laboratories (Rehovot, Israel). PCR reactions were performed in an Eppendorf (Hamburg, Germany) Thermal Cycler using REDTaq ready mix (Sigma–Aldrich) in 20-μl reaction volumes, according to the manufacturers’ instructions. The PCR thermal profile consisted of an initial denaturation for 5 min at 95°C, followed by 35 cycles of denaturation for 30 s at 95°C, annealing for 30 s at X°C, and elongation at 72°C for Y s (X and Y, annealing temperatures and elongation times, respectively; detailed in Supplementary Table S4). A final extension step was performed at 72°C for 5 min. Five microliters of PCR product were separated by electrophoresis at 120 V for 30 min on a 1% agarose gel in 0.5X Tris-acetate ethylenediaminetetraacetic acid (EDTA) buffer. Subsequently, gels were stained with ethidium bromide and the gel images were captured using the BioDoc-ItTM System (UVP, Opland, CA, USA).

### Statistical Analysis

Student’s *t*-test with Bonferroni adjustment was used for analyses of ENC values and G + C contents within ORFs. The statistical analyses were done using SciPy package for Python (Jones et al., 2014).

## Author Contributions

SB, TP, and NE-L conceived the research. SB, TP and RW supervised the students involved in this project. NE-L, DS, and MG carried out the sequence analysis of the M6 genome and the comparative analysis between strains M6 and AAC00-1. NE-L and DT-A conducted the PCR assays. DS and NE-L did the statistical analyses. GMDS performed the coverage analysis of FA and RFA fragments in *A. citrulli* strains. SB, TP, NE-L and DS planned the structure of the manuscript. NE-L and SB wrote the draft of the manuscript. All authors edited the manuscript and approved it.

## Conflict of Interest Statement

The authors declare that the research was conducted in the absence of any commercial or financial relationships that could be construed as a potential conflict of interest.

## Footnotes

1 http://www.clcbio.com

2 www.opgen.com

3 http://rast.nmpdr.org/

4 http://biocyc.org

5 http://www.python.org

6 http://www.geneious.com
