# Supplementary material for: Insights from the Genome Sequence of Acidovorax citrulli M6, a Group I Strain of the Causal Agent of Bacterial Fruit Blotch of Cucurbits
Source: Front Microbiol. 2016 Apr 6;7:430. doi: 10.3389/fmicb.2016.00430 (PMC4821854; doi:10.3389/fmicb.2016.00430)
Supplement: Supplementary file 1 [file Data_Sheet_1.PDF]

Supplementary Material

Insights from the genome sequence of *Acidovorax citrulli* M6, a group I strain of the causal agent of bacterial fruit blotch of cucurbits

Noam Eckshtain-Levi, Dafna Shkedy, Michael Gershovits, Gustavo Mateus Da Silva, Dafna Tamir-Ariel, Ron Walcott, Tal Pupko and Saul Burdman*

*** Correspondence:** Saul Burdman: saul.burdman@mail.huji.ac.il

Supplementary Figures

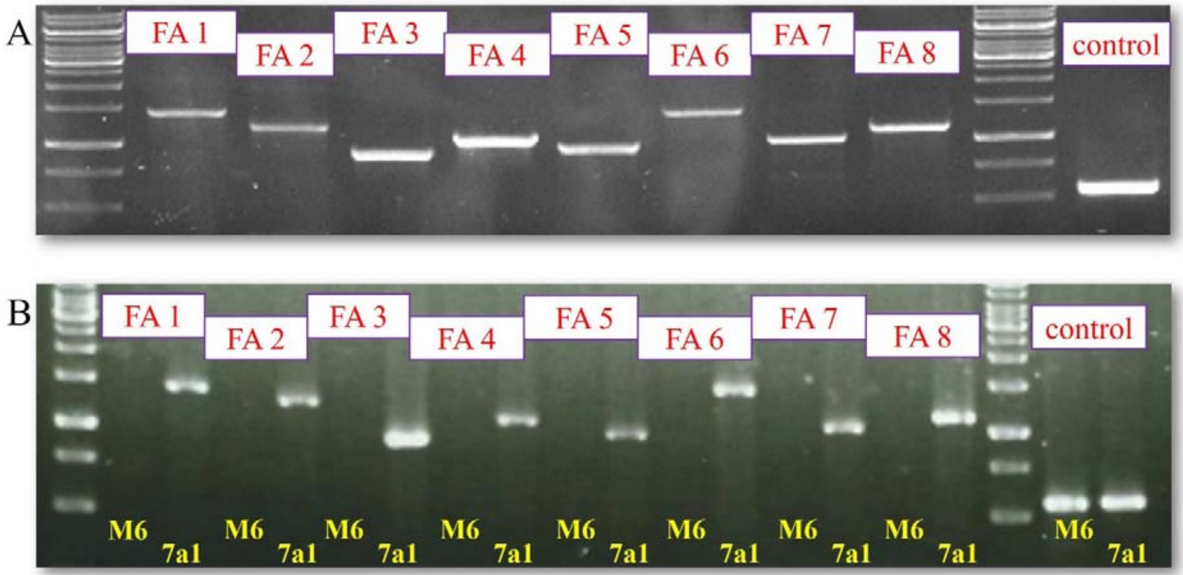

Supplementary Figure S1. PCR of the eight FA fragments and the housekeeping *gltA* gene (control) with DNA from *Acidovorax citrulli* strains. A, AAC00-1 (group II). B, M6 (group I) and 7a1 (group II). Lines: 1 Kb marker, FA fragments FA1 to FA8 of AAC00-1 (in A), and of M6 and 7a1 (in B), 1 Kb marker, and *gltA* product of AAC00-1 in A, and of M6 and 7a1 in B.

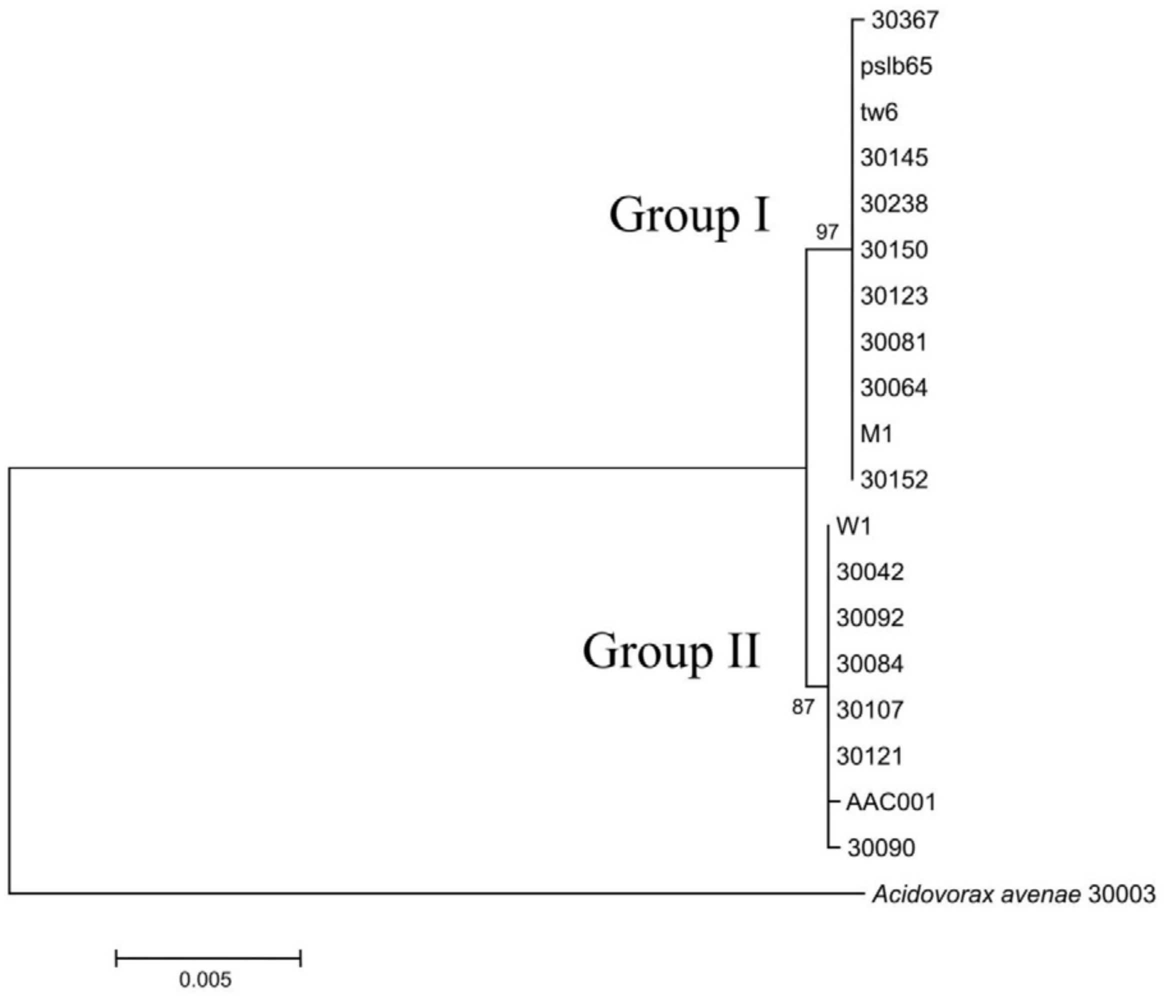

Supplementary Figure S2. Phylogenetic tree of *Acidovorax citrulli* strains based on seven housekeeping genes. Concatenated sequences from seven conserved genes (*gltA, trpB, lepA, ugpB, gmc, phaC* and *pilT*) were used to build a maximum likelihood phylogenetic tree with 1,000 replicates bootstrap test. The tree was modified from Eckshtain-Levi et al. (Eckshtain-Levi *et al.*, 2014), to include data from the two recently sequenced strains pslb65 and tw6 (Wang *et al.*, 2015a; Wang *et al.*, 2015b). These two strains clearly cluster with group I strains. *Acidovorax avenae* ICPB 30003 was used as outgroup.

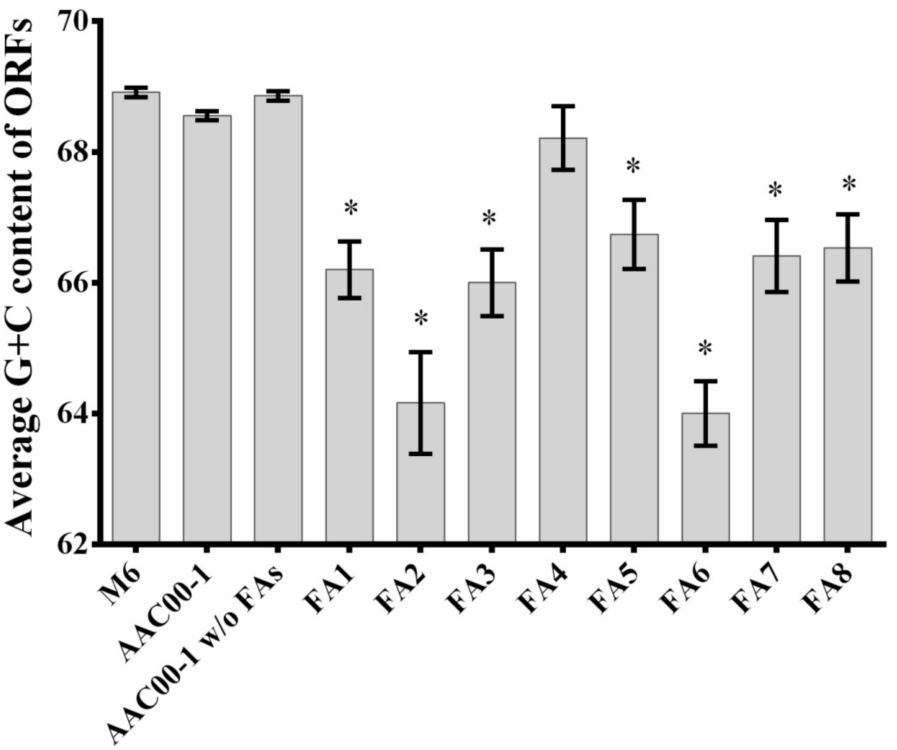

Supplementary Figure S3. Average G+C content within ORFs of *Acidovorax citrulli* strains M6 and AAC00-1, AAC00-1 without (w/o) the FA fragments, and each of the eight FA fragments. Data represent average G+C content of ORFs and standard errors. Asterisks indicate that the average G+C content value significantly ($p<0.05$) differs from the average G+C content value of AAC00-1 without the FAs, according to student's t-test with Bonferroni adjustment.

Supplementary Tables

Supplementary Table S1. List of universally conserved housekeeping genes used for quality control of the *Acidovorax citrulli* M6 sequence.

Protein name	Identity percentage (relative to AAC00-1) [a]	Amino acid length in M6	Percentage of coverage [b]
DnaG	100%	431	100%
Frr	100%	182	100%
InfC	100%	159	100%
NusA	100%	490	100%
Pgk [b]	99%	396	100%
PyrG	100%	548	100%
RplA	100%	231	100%
RplB	100%	274	100%
RplC	100%	211	100%
RplD	100%	195	100%
RplE	100%	178	100%
RplF	100%	177	100%
RplK	100%	141	100%
RplL	100%	126	100%
RplM	100%	142	100%
RplN	98%	122	100%
RplP	100%	136	100%
RplS	100%	115	100%
RplT	100%	118	100%
RpmA	100%	82	100%
RpoB	100%	1366	100%
RpsB	100%	245	100%
RpsC	100%	209	100%
RpsE	100%	160	100%
RpsI	100%	123	100%
RpsJ	100%	102	100%
RpsK	100%	134	100%
RpsM	100%	114	100%
RpsS	100%	92	100%
SmpB	100%	156	100%
Tsf	100%	283	100%

[a] The translated amino acid sequences of the corresponding genes were collected from *E. coli* K12. Then the amino acid sequences of the ortholog genes were retrieved from AAC00-1 by tBlastN. These

sequences were then used to screen the contigs of the M6 genome by tBlastN. Values show the level of identity between M6 and AAC00-1 at the amino-acid level.
[b] Coverage percentage between M6 and AAC00-1 at the amino acid level. All genes were fully covered in M6.

Supplementary Table S2. Length and coordinates of FA and RFA fragments used for coverage analyses [a].

Fragment number	Length (bp)	Start position [b]	End position [b]	GC content (%) [c]
FA 1	119,542	516,863	636,404	65.44
FA 2	34,954	742,913	777,866	63.08
FA 3	39,694	1,763,141	1,802,834	66.31
FA 4	48,243	1,897,321	1,945,563	67.17
FA 5	58,108	2,554,496	2,612,603	67.41
FA 6	110,748	2,940,586	3,051,333	63.88
FA 7	40,371	3,172,544	3,212,914	66.81
FA 8	41,587	4,544,572	4,586,158	66.67
RFA 1	30,495	264,275	294,769	68.88
RFA 2	40,408	661,024	701,431	67.92
RFA 3	70,882	1,051,409	1,122,290	67.83
RFA 4	52,298	1,830,605	1,882,902	66.56
RFA 5	72,856	2,137,455	2,210,310	69.96
RFA 6	70,342	2,702,315	2,772,656	66.78
RFA 7	72,517	3,087,884	3,160,400	68.69
RFA 8	121,263	3,809,041	3,930,303	68.27
RFA 9	101,161	4,725,210	4,826,370	69.07

[a] FA fragments, fragments that are present in the genome of AAC00-1 and absent in the genome of M6. RFA, reference fragments, present in both genomes (control fragments for coverage analysis).
[b] The start and end coordinates of each fragment, according to the AAC00-1 genome.
[c] G+C content of the fragments.

Supplementary Table S3. List of primers used to amplify regions of the FA fragments and part of the *gltA* gene.

Number	Primer name [a]	Sequence	Target genes in AAC00-1 [b]
1	Fragment 1_F	TGGAAAACGGTCTCAAGAAG	*Aave_0500* (h. p.) & *Aave_0501* (h. p.)
2	Fragment 1_R	GCGCTTCACTATCACCATCT	
3	Fragment 2_F	TCGGAAAGTTCTTTTGATCG	*Aave_0700* (major facilitator superfamily transporter) & *Aave_0701* (h. p.)
4	Fragment 2_R	GAGGAAACATCCGAACAATG	
5	Fragment 3_F	CTCATGCTGCGGATAGAGAT	*Aave_1619* (HK97 phage protein), *Aave_1620* (h. p.) & *Aave_1621* (h. p.)
6	Fragment 3_R	TCTCCCCAATGTAGAGCTTG	
7	Fragment 4_F	GAAGAAGAACCTTGCACAGC	*Aave_1760* (h. p.)
8	Fragment 4_R	CACGTAGTCGCGATAACTCC	
9	Fragment 5_F	CTGACGCTCATCGACATCAC	*Aave_2369* (h. p.), *Aave_2370* (h. p.) & *Aave_2371* (h. p.)
10	Fragment 5_R	GTTGATCGCAAATCCGTTG	
11	Fragment 6_F	CCGTTTTTGTTCTTGTCTTGA	*Aave_2759* (h. p.), *Aave_2760* (h. p.) & *Aave_2761* (h. p.)
12	Fragment 6_R	CTACGTCGAATCGCACAAC	
13	Fragment 7_F	AAGCTGCAGAAGTTCGTCAC	*Aave_2900* (phage protein)
14	Fragment 7_R	TCATCGTTGCTTTCTGAACA	
15	Fragment 8_F	AGGAAAACGTGATCAAGCAG	*Aave_4100* (h. p.)
16	Fragment 8_R	TTTTCCTACAATGCGGACAT	
17	Aave_2199_R	GAAGTCCACGTTCGGGTAGA	*Aave_2199* (housekeeping gene *gltA*, citrate synthase)
18	Aave_2199_F	TACATGTACCCGCAGAACCA	

[a] Numbers in primer names correspond to the FA number in AAC00-1 (Figure 1). F, forward; R, reverse.
[b] h. p., hypothetical protein.

Supplementary Table S4. Details of PCR reactions that were used to amplify segments using the primers listed in Supplementary Table S3.

Amplified fragment	Primers [a]	Annealing temperature (°C)	Elongation time (s)	Size (bp) of PCR product [c]
Fragment 1	1+2	52	90	1,417
Fragment 2	3+4	52	80	1,253
Fragment 3	5+6	52	60	921
Fragment 4	7+8	52	65	1,087
Fragment 5	9+10	53	60	969
Fragment 6	11+12	52	90	1,429
Fragment 7	13+14	52	65	1,026
Fragment 8	15+16	51	75	1,140
Aave_2199 [b]	17+18	54	30	489

[a] Primer numbers corresponding to Supplementary Table S3.

[b] Corresponds to part of the housekeeping gene *gltA*.

[c] Expected sizes of the PCR products according to the AAC00-1 annotation.

Supplementary Table S5. List of the 123 *Acidovorax citrulli* M6 genes that are absent in the genome of strain AAC00-1 based on comparative analysis between the genomes annotated by RAST.

Gene ID RAST [a]	Gene ID NCBI [b]	Function [c]	Coverage (%) [d]	
			pslb65	tw6
peg.9	APS58_00685	calpastatin	100	100
peg.64	APS58_07930	hypothetical protein	100	100
peg.87	APS58_03995	hypothetical protein	100	0
peg.88	APS58_04000	hypothetical protein	100	0
peg.89	APS58_04005	conjugal transfer protein TraC [f]	100	0
peg.90	APS58_04010	hypothetical protein	100	0
peg.140	APS58_02455	hypothetical protein	100	100
peg.198	APS58_11235	hypothetical protein	100	100
peg.205	APS58_11270	CopG protein	100	100
peg.215	APS58_11320	hypothetical protein	100	100
peg.221	APS58_11350	hypothetical protein	100	0
peg.222	APS58_11355	erythrocyte membrane protein 1	100	0
peg.226	APS58_11375	hypothetical protein	100	0
peg.228	APS58_11385	RND transporter [f]	100	0
peg.230	APS58_11395	hypothetical protein	100	0
peg.231	APS58_11400	arylsulfatase [f]	100	0
peg.232	APS58_11405	RND transporter [f]	100	0
peg.233	APS58_11410	glycosyl hydrolase	100	0
peg.234	APS58_11415	NADH dehydrogenase	100	0
peg.237	APS58_11430	3-(3-hydroxy-phenyl)propionate hydroxylase	100	0
peg.238	APS58_11435	NIPSNAP family protein [f]	100	0
peg.241	APS58_11450	glyoxalase	100	0
peg.242	APS58_11455	glycosyl hydrolase	100	0
peg.246	APS58_11475	hypothetical protein	100	0
peg.294	APS58_11715	hypothetical protein	100	100
peg.302	APS58_01115	hypothetical protein	100	0
peg.303	APS58_01120	hypothetical protein	100	0
peg.304	APS58_01125	IncQ plasmid conjugative transfer DNA nicking endonuclease TraR	100	0
peg.305	APS58_01130	stability protein StdB	100	0
peg.333	APS58_20735	hypothetical protein	100	0
peg.340	APS58_07105	hypothetical protein	100	100
peg.355	APS58_07180	hypothetical protein	100	100
peg.359	APS58_07200	hypothetical protein	100	100
peg.432	APS58_06950	hypothetical protein	100	97.67
peg.476	APS58_21070	isopentenyl transferase [f]	100	100
peg.837	APS58_12125	hypothetical protein	100	100
peg.861	APS58_12245	hypothetical protein	100	100
peg.892	APS58_12400	hypothetical protein	100	100

peg.964	APS58_12760	low-complexity acidic protein	100	100
peg.997	APS58_12925	hypothetical protein	100	100
peg.1079	APS58_13215	type IV secretion protein Rhs	100	100
peg.1168	APS58_09520	hypothetical protein	100	100
peg.1171	APS58_09535	hypothetical protein	100	100
peg.1200	APS58_04455	hypothetical protein	100	100
peg.1300	APS58_09720	hypothetical protein	100	100
peg.1423	APS58_00165	membrane protein [f]	100	0
peg.1424	APS58_00170	hypothetical protein	100	0
peg.1426	APS58_00180	cobalt ABC transporter permease [f]	100	0
peg.1427	APS58_00185	phage-related protein	100	0
peg.1428	APS58_00190	hypothetical protein	0	0
peg.1476	APS58_00430	head-tail connector protein [f]	100	100
peg.1510	APS58_00600	hypothetical protein	100	100
peg.1552	APS58_20205	diguanylate cyclase [f]	100	100
peg.1555	APS58_20220	methyltransferase [f]	100	0
peg.1558	APS58_10910	lead, cadmium, zinc and mercury transporting ATPase	100	100
peg.1568	APS58_06185	NinG protein [f]	100	100
peg.1569	APS58_06190	hypothetical protein	100	100
peg.1609	APS58_06390	Sel1 repeat protein [f]	100	0
peg.1680	APS58_07420	hypothetical protein	100	100
peg.1759	APS58_17470	cysteinyl-tRNA synthetase	100	0
peg.1760	APS58_17475	putative ATP-binding protein	100	13.81
peg.1772	APS58_17535	nucleoside diphosphate kinase	100	100
peg.1773	APS58_17540	hypothetical protein	100	100
peg.2042	APS58_05510	hypothetical protein	100	100
peg.2181	APS58_08860	hybrid sensor histidine kinase/response regulator [f]	100	100
peg.2185	APS58_08880	hypothetical protein	100	100
peg.2264	APS58_14570	probable nuclear antigen	100	100
peg.2308	APS58_13405	hypothetical protein	100	0
peg.2353	APS58_13630	hypothetical protein	100	0
peg.2370	APS58_13715	hypothetical protein	100	100
peg.2432	APS58_14025	hypothetical protein	100	100
peg.2478	APS58_14255	hypothetical protein	100	100
peg.2489	APS58_14310	serine/threonine protein kinase [f]	100	100
peg.2490	APS58_14315	hypothetical protein	100	100
peg.2511	APS58_03030	hypothetical protein	100	100
peg.2596	APS58_01370	hypothetical protein	100	100
peg.2624	APS58_01505	hypothetical protein	100	100
peg.2661	APS58_10350	hypothetical protein	100	100
peg.2704	APS58_02520	NACHT domain protein [f]	100	100
peg.2750	APS58_02750	hypothetical protein	100	100
peg.2762	APS58_02810	hypothetical protein	100	98.67
peg.2982	APS58_14410	hypothetical protein	100	100
peg.3034	APS58_06055	hypothetical protein	100	100
peg.3038	APS58_06075	hypothetical protein	100	100

peg.3236	APS58_06790	Putative two component system histidine kinase YedV	100	100
peg.3332	--[e]	hypothetical protein	100	100
peg.3378	--[e]	hypothetical protein	100	100
peg.3384	--[e]	hypothetical protein	100	100
peg.3430	--[e]	hypothetical protein	100	100
peg.3491	APS58_02015	diguanylate phosphodiesterase	100	99.68
peg.3515	APS58_10370	putative phage protein	0	0
peg.3534	APS58_01985	cell filamentation protein Fic	100	0
peg.3535	APS58_01990	plasmid replication protein [f]	100	0
peg.3537	APS58_02000	hypothetical protein	100	0
peg.3539	APS58_02010	hypothetical protein	100	0
peg.3711	APS58_05855	type IV secretion system protein VirB8/TraJ	100	0
peg.3717	APS58_05885	IncQ plasmid conjugative transfer DNA primase TraO	100	0
peg.3731	APS58_19940	hypothetical protein	100	100
peg.3768	APS58_03460	hypothetical protein	100	100
peg.3778	APS58_03510	hypothetical protein	100	100
peg.3788	APS58_03560	hypothetical protein	100	100
peg.3857	APS58_19855	hypothetical protein	100	100
peg.3876	APS58_02965	hypothetical protein	100	100
peg.3877	APS58_07465	TKL/LISK/LISK-DD1 protein kinase [f]	100	0
peg.3878	APS58_07470	hypothetical protein	100	0
peg.3880	APS58_07480	type IV secretion system protein virB3 [f]	100	0
peg.3881	APS58_07485	conjugal transfer protein [f]	100	0
peg.3882	APS58_07490	hypothetical protein	100	0
peg.3956	APS58_08320	2,3-butanediol dehydrogenase [f]	100	0
peg.3974	APS58_08410	cytosol aminopeptidase [f]	100	100
peg.4074	APS58_20025	hypothetical protein	100	100
peg.4077	APS58_02470	hypothetical protein	100	100
peg.4080	APS58_02485	hypothetical protein	100	100
peg.4122	APS58_14840	hypothetical protein	100	100
peg.4179	APS58_15125	dihydrolipoamide acetyltransferase	100	100
peg.4194	APS58_15200	hypothetical protein	100	100
peg.4224	APS58_15350	hypothetical protein	100	100
peg.4240	APS58_15430	xanthan lyase [f]	100	100
peg.4251	APS58_15485	hypothetical protein	100	100
peg.4364	APS58_19880	heat-shock protein [f]	100	0
peg.4365	APS58_00800	hemoglobin-like protein HbO	100	100
peg.4366	APS58_00805	mobile element protein	100	100
peg.4370	APS58_07505	hypothetical protein	98.85	0

[a] Gene ID according to the annotation at the RAST server.

[b] Gene ID according to the annotation at the NCBI server.

[c] Protein function as determined by automated Blast at the RAST server.

[d] Coverage percentage of the M6 genes in the two sequenced strains pslb65 and tw6 determined by BlastN.

[e] Genes annotated only by RAST.

[f] All open reading frames annotated as hypothetical proteins by RAST were manually analyzed by BlastP. In cases where putative functions were assigned by BlastP, these are indicated followed by "[f]".
